# Association Between Previous or Active Cancer and Clinical Outcomes in TAVR Patients: A Systematic Review and Meta-Analysis of 255,840 Patients

**DOI:** 10.3389/fcvm.2021.763557

**Published:** 2021-11-02

**Authors:** Carlos Diaz-Arocutipa, Javier Torres-Valencia, Gabriela Zavaleta-Camacho, Lourdes Vicent

**Affiliations:** ^1^Vicerrectorado de Investigación, Universidad San Ignacio de Loyola, Lima, Peru; ^2^Programa de Atención Domiciliaria – EsSalud, Lima, Peru; ^3^Departamento de Cardiología, Hospital Nacional Edgardo Rebagliati Martins, Lima, Peru; ^4^Facultad de Medicina Alberto Hurtado, Universidad Peruana Cayetano Heredia, Lima, Peru; ^5^Cardiology Department, Hospital Universitario 12 de Octubre, Madrid, Spain; ^6^Instituto de Investigación Sanitaria Hospital 12 de Octubre (imas12), Madrid, Spain

**Keywords:** aortic stenosis, TAVR, cancer, systematic review, mortality

## Abstract

**Background:** It has been proposed that transcatheter aortic valve replacement (TAVR) may be an option for patients with cancer and severe aortic stenosis. We assessed the association between previous or active cancer and clinical outcomes in TAVR patients.

**Methods:** We searched four electronic databases from inception to March 05, 2021. The primary outcome was all-cause mortality. Secondary outcomes were cardiovascular mortality, myocardial infarction, stroke, acute kidney injury, pacemaker implantation, major bleeding, and vascular complications. All meta-analyses were performed using a random-effects model. Relative risks (RRs) and adjusted hazard ratios (aHRs) with their 95% confidence interval (95% CI) were pooled.

**Results:** Thirteen cohort studies involving 255,840 patients were included. The time period for mortality ranged from inpatient to 10 years. Patients with active cancer had a higher risk of all-cause mortality using both crude (RR, 1.46; 95% CI, 1.13–1.88) and adjusted (aHR, 1.79; 95% CI, 1.43–2.25) estimates compared to non-cancer group. In contrast, the risk of cardiovascular mortality (RR, 1.26; 95% CI, 0.58–2.73), myocardial infarction (RR, 0.94; 95% CI, 0.34–2.57), stroke (RR, 0.90; 95% CI, 0.75–1.09), pacemaker implantation (RR, 0.87; 95% CI, 0.50–1.53), acute kidney injury (RR, 0.88; 95% CI, 0.74–1.04), major bleeding (RR, 1.15; 95% CI, 0.80–1.66), and vascular complications (RR, 0.96; 95% CI, 0.79–1.18) was similar between patients with or without cancer.

**Conclusion:** Our review shows that TAVR patients with active cancer had an increased risk of all-cause mortality. No significant association with secondary outcomes was found.

## Introduction

Transcatheter aortic valve replacement (TAVR) has become a safe and effective treatment option for patients with symptomatic severe aortic stenosis (1). It is well-known that a large proportion of patients undergoing TAVR are elderly with multiple comorbidities that may influence their short-term prognosis (1). Among them, it has been estimated that ~20% of TAVR patients have a history of cancer (2). However, cancer patients have often been excluded from pivotal TAVR trials. Given their likely poor survival, the decision as to whether a patient with cancer and severe aortic stenosis is a candidate for TAVR is complex. Moreover, severe aortic stenosis could potentially condemn patients to a higher mortality risk than cancer itself, and access to TAVR may provide a longer life expectancy.

Several studies with mixed results have been reported evaluating the association between cancer and outcomes in TAVR patients (2–5). Moreover, it is unknown whether the outcomes vary if the patients had previous cancer or it was active. Therefore, we performed a systematic review and meta-analysis to assess the association between previous or active cancer and clinical outcomes in patients with severe aortic stenosis treated with TAVR.

## Methods

This systematic review was reported according to the 2009 PRISMA (Preferred Reporting Items for Systematic Reviews and Meta-analyses) statement (6).

### Search Strategy

We searched in four electronic databases (PubMed, Embase, Web of Science, and Scopus) from inception to March 05, 2021. The complete search strategy can be found in Supplementary Table 1. There were no restrictions on publication year or language. The reference lists of included studies and relevant reviews were also screened to identify eligible studies.

### Eligibility Criteria

The inclusion criteria were the following: (i) cohort studies evaluating the association between previous or active cancer and clinical outcomes in adult patients (≥18 years old) with severe aortic stenosis treated with transcatheter aortic valve replacement and (ii) studies reporting at least one of the primary or secondary outcomes at any length of follow-up. We only included cohort studies since it is not possible to evaluate cancer as exposure in randomized controlled trials. Cross-sectional studies, case-control studies, case series, case reports, systematic reviews, conference abstracts, and editorials were excluded.

### Study Selection

All articles from the search were downloaded and duplicates were removed. Title/abstract and full-texts were independently assessed by two review authors (JTV and GZ). Any disagreement was resolved by a third review author (CDA).

### Outcomes

The primary outcome was all-cause mortality. Secondary outcomes were cardiovascular mortality, myocardial infarction, stroke, pacemaker implantation, acute kidney injury, major bleeding, and vascular complications. The study definitions were used for all outcomes.

### Data Extraction

Two authors (JTV and GZ) independently extracted the information from each study using a standard data extraction form that was previously piloted. Any disagreement was resolved by a third review author (CDA). The following data were extracted: first author name, year of publication, country, study design, type of population, sample size, age, sex, comorbidities, the timing of cancer, follow-up duration, and primary and secondary outcomes.

### Risk of Bias Assessment

Two review authors (CDA and JTV) independently assessed the risk of bias of each cohort study using the Newcastle-Ottawa Scale (NOS) tool (7). Any disagreement was resolved by consensus. The NOS tool rates cohort studies based on three domains: selection, comparability, and outcome. The selection domain consists of four items: representativeness of the exposed cohort, selection of the non-exposed cohort, ascertainment of exposure, and demonstration that the outcome of interest was not present at the start of the study. The comparability domain consists of one item: comparability of cohorts on the basis of the design or analysis. The outcome domain consists of three items: assessment of outcome, was follow-up long enough for outcomes to occur, and adequacy of follow-up of cohorts. Each item is scored with zero, one, or two stars. Overall, each study was judged as follows: low risk of bias (8–9 stars), moderate risk of bias (5–7 stars), and high risk of bias (0–4 stars) (8).

### Statistical Analysis

All meta-analyses were performed using a random-effects model. The between-study variance (tau^2^) was estimated using the Paule-Mandel method (9). Unadjusted relative risks (RRs) and adjusted hazard ratios (aHRs) with their 95% confidence intervals (CIs) were pooled. We have combined the aHRs from each study as reported. Statistical heterogeneity was evaluated using the Chi-square test (*p* < 0.10 as threshold) and the I^2^ statistic (10). Heterogeneity was defined as follows: low if *I*^2^ <30, moderate if *I*^2^ = 30–60%, and high if *I*^2^ > 60%. Publication bias was assessed using the visual inspection of funnel plots and Egger's test if 10 or more studies were available (10). Subgroups analyses were performed according to the timing of cancer (previous history of cancer or active cancer). The test for subgroup differences (interaction test) was considered statistically significant if the *p*-value was <0.10 as previously recommend (10, 11). For the main meta-analyses, a two-tailed *p* < 0.05 was used for statistical significance. We used the package meta from R 3.6.3 (www.r-project.org) for all meta-analyses.

## Results

### Study Selection

**Figure 1:**
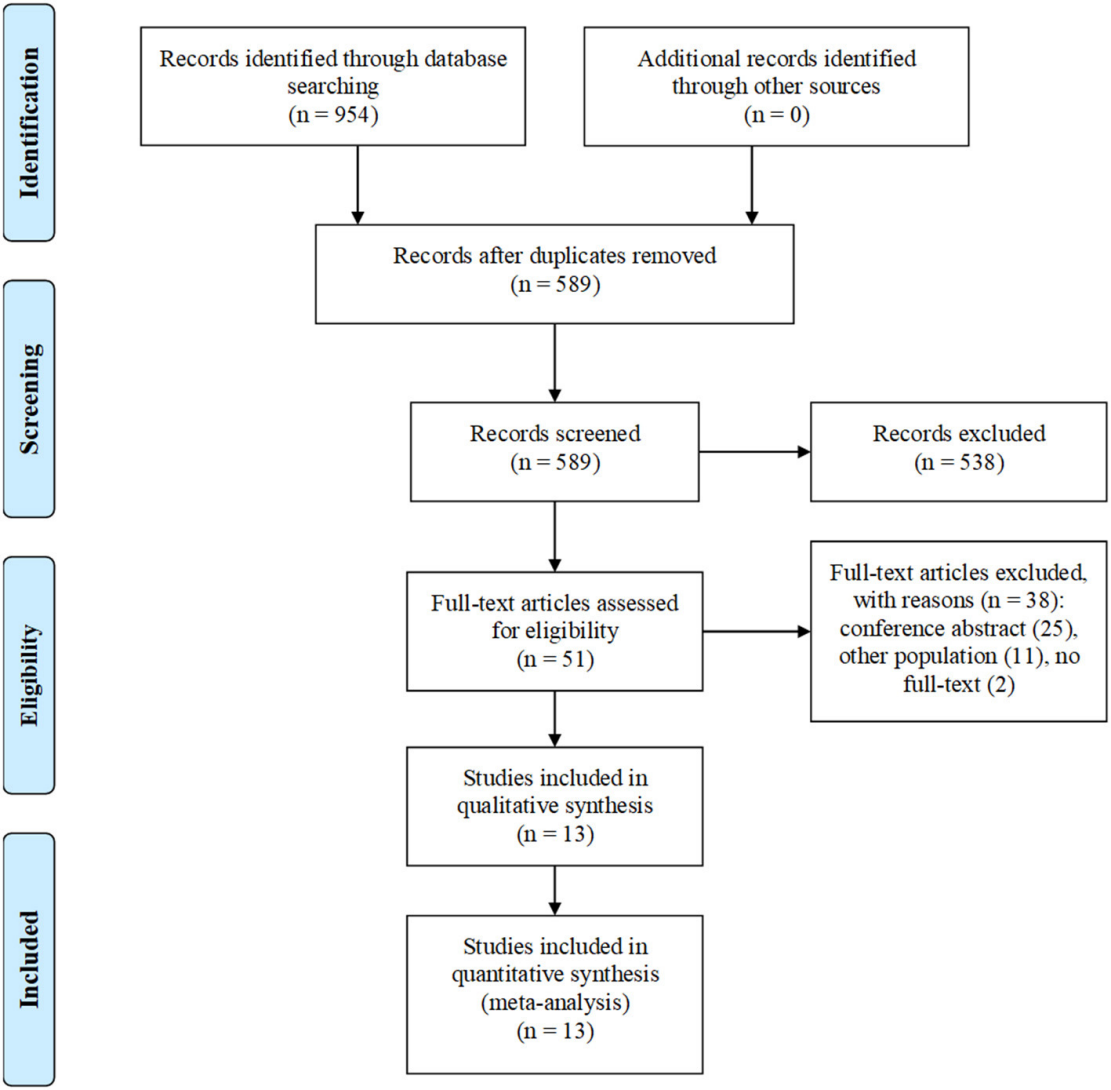
Flow diagram of study selection.

Our electronic search retrieved 954 articles. After the removal of 365 duplicates, 589 articles underwent title/abstract screening, of which 51 articles were included in the full-text screening. Finally, a total of 13 studies were selected (Figure 1) (2–5, 12–20).

### Study Characteristics

The main characteristics of the 13 cohort studies (*n* = 255.840 patients) are shown in Table 1. Eight studies were retrospective, four were prospective, and one was ambispective. Most studies were conducted in the United States of America (*n* = 4) and Germany (*n* = 3). The mean age ranged from 78.5 to 83 years and 50% were men. The most common comorbidities were hypertension (85%), dyslipidemia (60%), coronary artery disease (44%), and diabetes (32%). Active cancer was assessed in five studies and previous cancer in 10 studies. The prevalence of cancer in TAVR patients ranged from 4.5 to 22.9% across studies. The follow-up ranged from 1 month to 10 years. The median Society of Thoracic Surgeons (STS) score ranged from 3 to 8.1 across eight studies. The type of transcatheter aortic valve device was reported in six studies. The use of a self-expandable valve varied from 18 to 76% and a balloon-expandable valve from 24 to 100%. The access route of TAVR ranged from 82 to 100% across six studies. Information on the type of cancer was available in 10 studies (Supplementary Table 2). The most frequent cancer types were hematologic (25%), breast (22%), prostate (21%), and lung (18%). Only four studies reported data on cancer staging and treatment (Supplementary Table 2). The proportion of patients with metastases ranged from 6 to 31% across studies. Antineoplastic treatment was reported in 29–83% of cases. The adjusted effect estimates and adjusted variables of each study are described in Supplementary Table 3. The adjusted variables were not uniform across studies. The variables most commonly adjusted were age, sex, New York Heart Association scale, STS score, and coronary artery disease. None of the effect estimates were adjusted for time.

**Table 1:** Characteristics of included studies.

**References**	**Country**	**Study design**	**Population**	**Timing of cancer**	**Prevalence of cancer**	**Follow-up**	**Group**	**Sample size**	Age, years^*^	**Male**
Watanabe et al. (20)	Japan	Prospective cohort	Patients with symptomatic severe AS with NYHA II or greater undergoing TAVR	Active	6.3%	272 (142.5-401.5) days	Cancer	47	83 (80–87)	45%
							No cancer	702	85 (82–88)	33%
Agrawal et al. (12)	USA	Retrospective cohort	Patients with symptomatic severe AS who underwent TAVR	Previous	12.3%	17.1 months	Cancer	75	82 ± 8	39%
							No cancer	535	83 ± 8	54%
Biancari et al. (13)	Finland	Retrospective cohort	Patient with AS with or without coronary revasculariation undergoing TAVR	Previous	19.6%	2.1 ± 1.7 years	Cancer	417	80.6 ± 6.6	49%
							No cancer	1713	81.4 ± 6.6	44%
Grant et al. (2)	USA	Retrospective cohort	Adult patients with severe AS undergoing TAVR	Previous	19.2%	NR	Cancer	23,670	81.1 ± 7.9	57%
							No cancer	99,400	80.1 ± 6.7	53%
Guha et al. (3)	USA	Retrospective cohort	Hospitalized adults with severe AS undergoing TAVR	Previous	22.5%	NR	Cancer	10,670	81.1 ± 0.2	57%
							No cancer	36,625	80.6 ± 0.1	53%
Jain et al. (15)	USA	Retrospective cohort	Patient with severe AS undergoing TAVR	Active	4.5%	30 days	Cancer	2,849	83 (76–87)	61%
							No cancer	60,503	83 (77–88)	52%
Ghotra et al. (14)	USA	Retrospective cohort	Adults patients with severe AS who underwent TAVR	Previous	16.7%	1 year	Cancer	181	NR	NR
							No cancer	900		
Landes et al. (4)	Various countries	Ambispective cohort	Patients who undergo TAVR while having active malignancy	Active	8.1%	330 (118-656) days	Cancer	222	78.8 ± 7.5	62%
							No cancer	2,522	81.3 ± 7.1	45%
Lantelme et al. (5)	France	Retrospective cohort	Adult hospitalized patients with AS undergoing TAVR	Previous	20%	2.09 ± 1.36 years	Cancer	2,050	82.5 ± 7	50%
							No cancer	8,171		
Lind et al. (16)	Germany	Prospective cohort	Consecutive patients included in their dedicated local registry for transfemoral TAVR	Active/ previous	22.9%	10 years	Active cancer	53	78.5 ± 6.4	45%
							Stable cancer	196	81.8 ± 5.6	52%
							No cancer	839	81.4 ± 5.4	46%
Mangner et al. (17)	Germany	Prospective cohort	Patients with severe AS treated with a transfemoral TAVR	Active/previous	19.2%	12 months	Active cancer	99	81 (77–84)	60%
							Tumor disease	251	80 (76–84)	42%
							No cancer	1,471	81 (77–84)	43%
Romeo et al. (18)	Argentina	Retrospective cohort	Patients with severe AS undergoing transfemoral TAVR	Previous	20.7%	12 months	Cancer	23	85 (80–88)	43%
							No cancer	88		
Tabata et al. (19)	Germany	Prospective cohort	Consecutive patients with severe AS undergoing TAVR	Previous (85%)	6.3%	5 years	Cancer	298	80.8 ± 5.8	61%
							No cancer	1,270	81.1 ± 6.7	48%

*CV, cardiovascular; MI, myocardial infarction; AKI, acute kidney injury; AS, aortic stenosis; TAVR, transcatheter aortic valve replacement; NR, not reported*.

* *Data are presented as mean ± standard deviation or median (interquartile range)*.

### Risk of Bias Assessment

According to the NOS tool, eight studies were scored as low risk of bias and five studies as the moderate risk of bias (Supplementary Table 4).

### All-Cause Mortality

**Figure 2:**
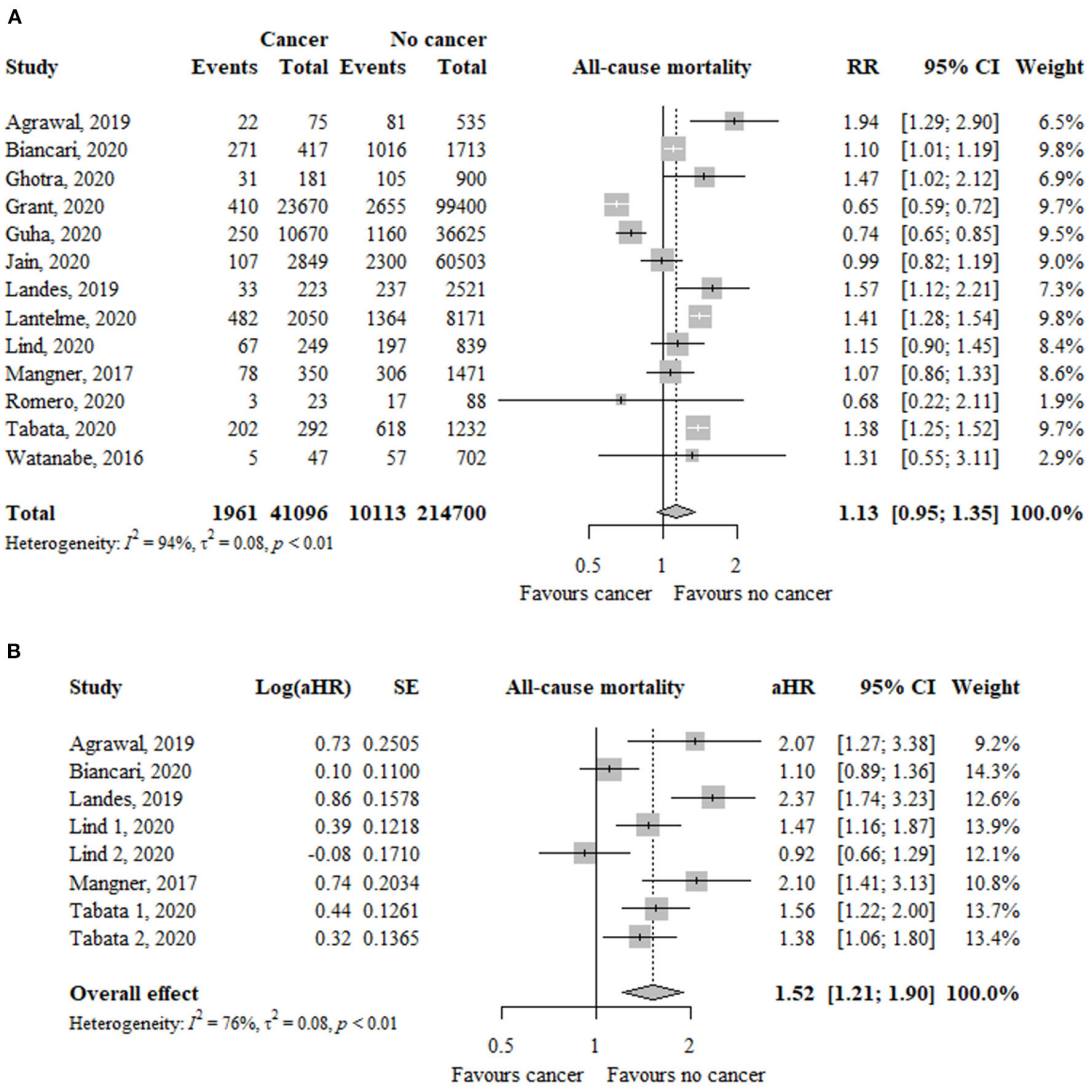
Association between cancer and all-cause mortality in TAVR patients using **(A)** risk ratios and **(B)** adjusted hazard ratio as effect measures. RR, relative risk; aHR, adjusted hazard ratio; CI, confidence interval; TAVR, transcatheter aortic valve replacement.

In 13 studies (*n* = 255.796), the risk of all-cause mortality was similar between patients with and without cancer (RR, 1.13; 95% CI, 0.95–1.35; *I*^2^ = 94%) (Figure 2). The funnel plot did not show asymmetry and the Egger's test was not significant (*p* = 0.68) (Supplementary Figure 1).

In eight studies (*n* = 9.917), using adjusted estimates, the risk of all-cause mortality was significantly higher in the cancer group compared to the non-cancer group (aHR, 1.52; 95% CI, 1.21–1.90; *I*^2^ = 76%) (Figure 2).

Only patients with active cancer, but no previous cancer, had a significantly increased risk of all-cause mortality using unadjusted (RR, 1.46; 95% CI, 1.13–1.88; *I*^2^ = 79%) and adjusted (aHR, 1.79; 95% CI, 1.43–2.25; *I*^2^ = 59%) effect estimates (Table 2). The test for subgroup differences suggests that there is a statistically significant subgroup effect using unadjusted (*p* = 0.06) and adjusted (*p* = 0.08) effect estimates.

**Table 2:** Subgroup analyses according to the timing of cancer.

**Outcomes**	**Number of**	**Effect**	**95% CI**	***I* ^**2**^**	***p*-value for**
	**studies**	**measures**			**interaction**
**All-cause mortality**					
Previous cancer	10	RR: 1.06	0.84–1.32	95%	0.06
Active cancer	5	RR: 1.46	1.13–1.88	79%	
**All–cause mortality (aHR)**					
Previous cancer	4	aHR: 1.26	0.92–1.73	66%	0.08
Active cancer	4	aHR: 1.79	1.43–2.25	59%	
**Cardiovascular mortality**					
Previous cancer	2	RR: 1.65	0.66–4.18	90%	0.13
Active cancer	2	RR: 0.50	0.14–1.75	0%	
**Myocardial infarction**					
Previous cancer	4	RR: 0.75	0.35–1.60	0%	0.20
Active cancer	2	RR: 1.92	0.57–6.45	24%	
**Stroke**					
Previous cancer	6	RR: 0.96	0.71–1.31	66%	0.66
Active cancer	5	RR: 0.88	0.69–1.12	0%	
**Acute kidney injury**					
Previous cancer	4	RR: 0.82	0.67–1.00	88%	<0.01
Active cancer	5	RR: 1.10	1.01–1.18	0%	
**Pacemaker implantation**					
Previous cancer	6	RR: 0.84	0.38–1.88	97%	0.56
Active cancer	5	RR: 1.08	0.82–1.43	62%	
**Major bleeding**					
Previous cancer	5	RR: 0.95	0.81–1.12	68%	0.45
Active cancer	4	RR: 1.26	0.62–2.58	80%	
**Vascular complications**					
Previous cancer	3	RR: 1.07	0.92–1.25	0%	0.35
Active cancer	4	RR: 0.91	0.65–1.25	51%	

*CI, confidence interval; RR, risk ratio; aHR, adjusted hazard ratio*.

### Cardiovascular Mortality

**Figure 3:**
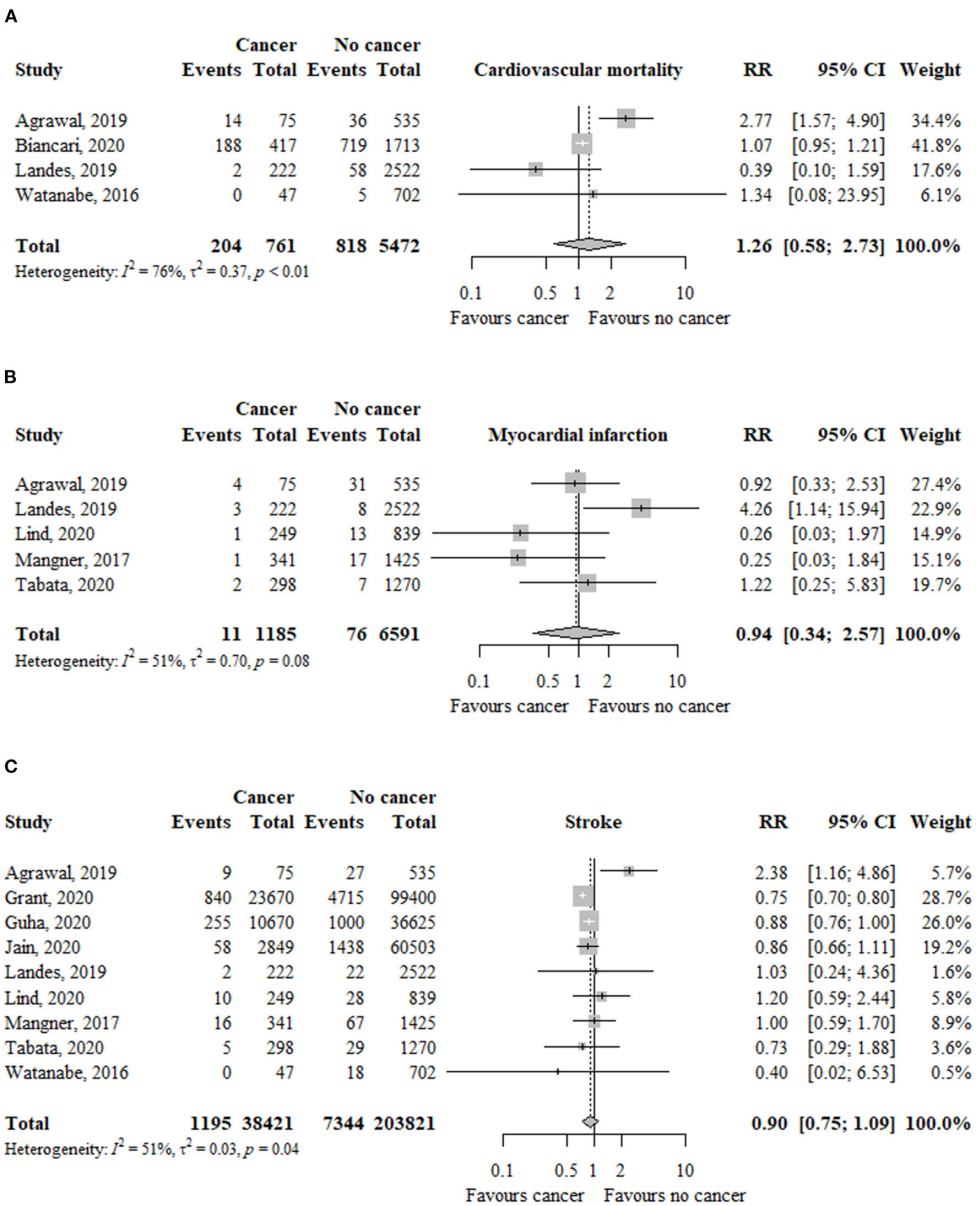
Association between cancer and **(A)** cardiovascular mortality, **(B)** myocardial infarction, and **(C)** stroke in TAVR patients. RR, relative risk; aHR, adjusted hazard ratio; CI, confidence interval; TAVR, transcatheter aortic valve replacement.

In four studies (*n* = 6.233), the risk of cardiovascular mortality was not significantly different between patients with and without cancer (RR, 1.26; 95% CI, 0.58–2.73; *I*^2^ = 76%) (Figure 3).

The risk of cardiovascular mortality was similar among patients with previous (RR, 1.65; 95% CI, 0.66–4.18; *I*^2^ = 90%) or active (RR, 0.50; 95% CI, 0.14–1.75; *I*^2^ = 0%) cancer compared to patients without cancer (Table 2). The test for subgroup differences was not significant (*p* = 13).

### Myocardial Infarction

In five studies (*n* = 7.776), the risk of myocardial infarction was not significantly different between patients with and without cancer (RR, 0.94; 95% CI, 0.34–2.57; *I*^2^ = 51%) (Figure 3).

The risk of myocardial infarction was not significantly different between patients with previous (RR, 0.75; 95% CI, 0.35–1.60; *I*^2^ = 0%) or active (RR, 1.92; 95% CI, 0.57–6.45; *I*^2^ = 24%) cancer compared to patients without cancer (Table 2). The test for subgroup differences was not significant (*p* = 20).

### Stroke

In nine studies (*n* = 242.242), the risk of myocardial infarction was similar between patients with and without cancer (RR, 0.90; 95% CI, 0.75–1.09; *I*^2^ = 51%) (Figure 3).

The risk of stroke was similar between patients with previous (RR, 0.96; 95% CI, 0.71–1.31; *I*^2^ = 66%) or active (RR, 0.88; 95% CI, 0.69–1.12; *I*^2^ = 0%) cancer compared to patients without cancer (Table 2). The test for subgroup differences was not significant (*p* = 66).

### Acute Kidney Injury

**Figure 4:**
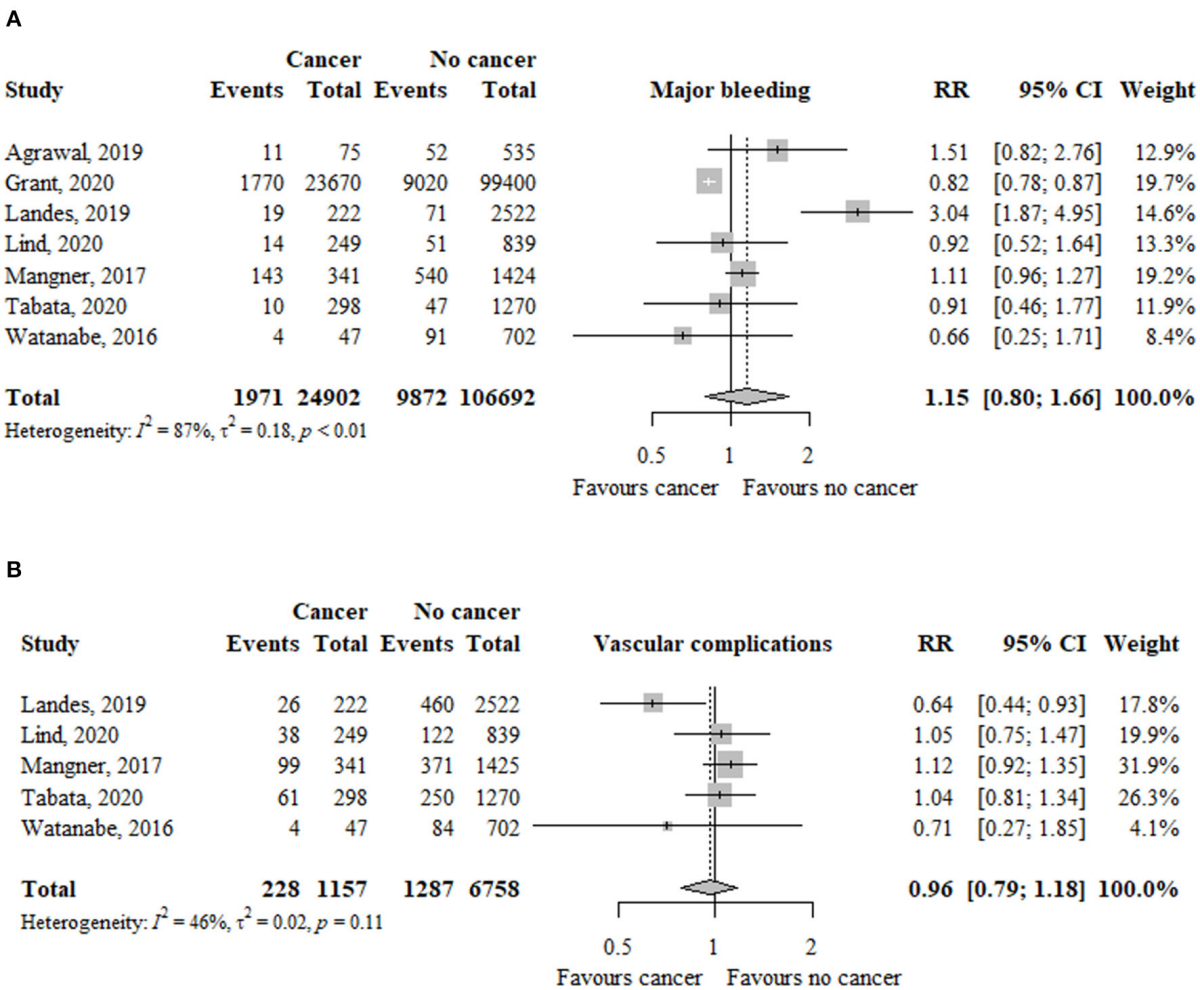
Association between cancer and **(A)** acute kidney injury and **(B)** pacemaker implantation in TAVR patients. RR, relative risk; aHR, adjusted hazard ratio; CI, confidence interval; TAVR, transcatheter aortic valve replacement.

In seven studies (*n* = 240.073), the risk of acute kidney injury was not significantly different between patients with and without cancer (RR, 0.88; 95% CI, 0.74–1.04; *I*^2^ = 94%) (Figure 4).

The risk of acute kidney injury was significantly higher in patients with active cancer (RR, 1.10; 95% CI, 1.01–1.18; *I*^2^ = 0%), but no previous cancer (RR, 0.82; 95% CI, 0.67–1.00; *I*^2^ = 88%), compared to patients without cancer (Table 2). The test for subgroup differences was significant (*p* < 0.01).

### Pacemaker Implantation

In nine studies (*n* = 244.987), the risk of pacemaker implantation was similar between patients with and without cancer (RR, 0.87; 95% CI, 0.50–1.53; *I*^2^ = 96%) (Figure 4).

The risk of pacemaker implantation was similar between patients with previous (RR, 0.84; 95% CI, 0.38–1.88; *I*^2^ = 97%) or active (RR, 1.08; 95% CI, 0.82–1.43; *I*^2^ = 62%) cancer compared to patients without cancer (Table 2). The test for subgroup differences was not significant (*p* = 56).

### Major Bleeding

**Figure 5:**
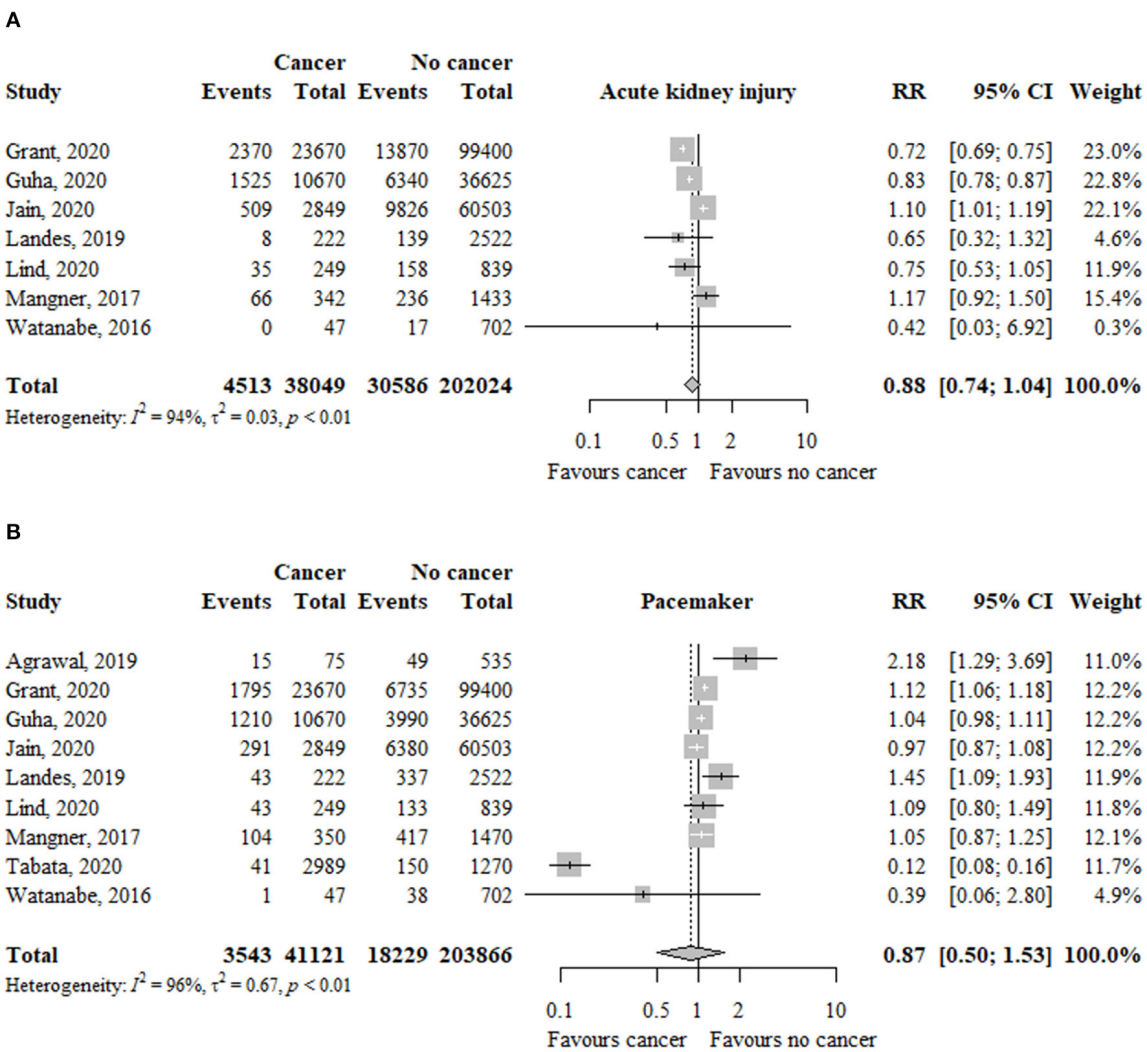
Association between cancer and **(A)** major bleeding and **(B)** vascular complications in TAVR patients. RR, relative risk; aHR, adjusted hazard ratio; CI, confidence interval; TAVR, transcatheter aortic valve replacement.

In seven studies (*n* = 131.594), the risk of major bleeding was not significantly different between patients with and without cancer (RR, 1.15; 95% CI, 0.80–1.66; *I*^2^ = 87%) (Figure 5).

The risk of major bleeding was not significantly different between patients with previous (RR, 0.95; 95% CI, 0.81–1.12; *I*^2^ = 97%) or active (RR, 1.26; 95% CI, 0.62–2.58; *I*^2^ = 80%) cancer compared to patients without cancer (Table 2). The test for subgroup differences was not significant (*p* = 45).

### Vascular Complications

In five studies (*n* = 7.915), the risk of vascular complications was similar between patients with and without cancer (RR, 0.96; 95% CI, 0.79–1.18; *I*^2^ = 46%) (Figure 5).

The risk of vascular complications was similar between patients with previous (RR, 1.07; 95% CI, 0.92–1.25; *I*^2^ = 0%) or active (RR, 0.91; 95% CI, 0.65–1.25; *I*^2^ = 51%) cancer compared to patients without cancer (Table 2). The test for subgroup differences was not significant (*p* = 35).

## Discussion

### Main Findings

In the present meta-analysis, we provide a comprehensive overview of the association between previous or active cancer and mortality and TAVR complications in 255.840 patients with severe aortic stenosis who underwent TAVR from 13 cohort studies. The main study findings can be summarized as follows: (1) only TAVR patients with active cancer had an increased risk of all-cause mortality using unadjusted and adjusted effect estimates; (2) the association between cancer (either previous or active) and cardiovascular mortality in patients who underwent TAVR was not significant; (3) complications after TAVR, such as acute myocardial infarction, need for pacemaker implantation, major bleeding and vascular complications, occurred similarly in cancer patients regardless of cancer activity, as in those without cancer.

### Association Between Cancer and Mortality After TAVR

Cancer is an increasingly frequent comorbidity in patients with cardiovascular diseases, with shared risk factors, such as obesity, a processed diet, smoking, or physical inactivity (21). This is also the case of aortic stenosis, the most common valve disease (22). In addition, cancer treatments, especially chest radiation therapy (23, 24), pose a specific risk for the development of aortic valve disease. Therefore, it is to be expected that in the coming years we will often have to deal with patients with severe aortic stenosis and cancer and make important decisions regarding the treatment of both entities. Latency to the presentation of valvular heart disease from cancer therapies is often over 20 years (23), so many patients with cancer have an inactive or stabilized tumor disease.

The contributions of this meta-analysis are relevant since, in patients with severe aortic stenosis who underwent TAVR implantation, only those who had active cancer had higher mortality from all causes. Patients with cancer present a higher risk of mortality and more life-threatening health conditions than the general population. The higher mortality has been attributed to both cardiovascular and non-cardiovascular conditions (25), but recently particular attention has been paid to cardiovascular disease-related deaths (26, 27), especially in certain subpopulations of cancer patients (26–28). As it has been described, mortality in cancer patients is strongly conditioned by the type of tumor (29). For those cancers of high malignancy (i.e., lung or pancreas), mortality is more likely due to cancer itself, but in others such as prostate, intestinal, or breast cancer, they present a high risk of mortality not attributable to cancer (29). In cancer patients over 60 years old, cardiovascular diseases are the main cause of death (29, 30). This elderly population is more prone to develop severe aortic stenosis, which can be more lethal than many malignancies if left untreated (31). Gastrointestinal (mainly colorectal cancer), breast, and prostate cancer were the most common malignancies in patients with severe aortic stenosis treated with TAVR (4, 13, 14, 17), and these tumors appear at older ages and tend to have a more indolent progression. Indeed, a study showed that breast cancer and prostate cancer were not associated with an increased risk of all-cause mortality (13). Regarding non-cardiac causes of death in patients with active cancer, the more commonly described are cerebrovascular disease, infections, liver failure, kidney disease (29), and cancer-related mortality, which represents ~50% of cancer patient's deaths (especially in those with progressive malignancies in stage III to IV) (4, 17). TAVR can be a reasonable option in patients with previous cancer, considering the stage of initial cancer and the duration of remission. In patients with active cancer there a comprehensive multidisciplinary assessment aimed to select candidates for TAVR should be conducted, given their higher risk of mortality in the mid- and long-term.

After relieving the aortic stenosis with the TAVR procedure, cardiovascular mortality was equaled in the three groups of patients (non-cancer, previous or active cancer), regardless of the history of cancer. The importance of managing aortic valve disease in cancer patients is that TAVR would allow a treatment directed to cancer with surgery, chemotherapy, targeted cancer therapies, or biological anticancer drugs that would improve the malignancy prognosis (4). In any case, it seems clear that an adequate selection of candidates for TAVR allows good cardiovascular results even in patients with active cancer, who may achieve a reasonable life expectancy after having solved a serious treatable disease, such as aortic stenosis, with a procedure with manageable complications.

### Association Between Cancer and TAVR Complications

This large-scale analysis allowed us to examine the association between cancer history and main TAVR complications. The risk of post-procedural complications was very similar in patients with active/previous cancer and non-cancer patients. However, it should be noted that information was scarce for some outcomes, limiting the robustness of the effect estimates. Only acute kidney injury occurred more frequently in patients with active cancer. Acute kidney injury is a common complication after TAVR, which may occur in half of the cases (although incidence varies widely) (32). Acute kidney injury after TAVR is multifactorial in origin: administration of iodinated contrast agents, bleeding and anemia, microembolisms, hypotension, or nephrotoxic drugs, among others. In addition, predisposing factors such as chronic kidney disease or previous heart failure play a role (32, 33). Although cancer patients tend to be younger and have fewer comorbidities (2), there are several cancer-related mechanisms underlying the higher risk of acute kidney injury, including a number of conventional chemotherapeutic agents, tumor infiltration, immune response, or volume depletion, among others (34).

The type of cancer may also play a role in the risk of post-TAVR acute kidney injury (3). The importance of this finding is that acute kidney injury after TAVR is associated with higher mortality, especially in those patients who develop stage III acute kidney injury (33), so preventive measures aimed at avoiding or minimizing kidney damage should be established early in cancer patients, especially those with active disease avoiding dehydration and withdrawing possible nephrotoxic drugs in the peri-intervention period.

In relation to other post-TAVR complications (stroke, pacemaker implantation, acute myocardial infarction, or bleeding), the risk was low and similar in the three groups of patients (without cancer, with active or previous cancer) (2–4, 12, 16, 17, 19, 20).

### Limitations

Our review has some limitations. First, since all included studies were observational and most were retrospective, there is a risk of confounding bias. Although we pooled unadjusted and adjusted effect estimates, there is a risk of residual confounding. Second, our findings are not extensible to patients treated with surgical aortic valve replacement as age and surgical risks are different from TAVR patients. Third, the heterogeneity was high among studies. Possible reasons include different sample sizes, heterogeneous definitions of bleeding and acute kidney injury, different types of cancer, and various lengths of follow-up. Fourth, information on the type, stage, and treatment of cancer was poorly reported across studies. Thus, it was not possible to assess the impact of these known prognostic factors on all-cause mortality. Fifth, since only a few studies were available for meta-analyses of some outcomes (e.g., cardiovascular mortality, myocardial infarction, and vascular complications) and their subgroups, pooled effect estimates for these outcomes should be interpreted with caution. Finally, it should be taken into account that possibly in patients with active cancer referred for TAVR implantation there may be a bias related to the prognosis of the malignancy itself since valve replacement would not have been considered in those with a very reduced life expectancy, or those in whom cancer treatment is not feasible. Therefore, life expectancy may have been overestimated in some patients with active cancer.

## Conclusion

Our meta-analysis shows that cancer patients present similar cardiovascular outcomes and post-procedural complications after TAVR. In patients with previous stable cancer, the overall prognosis is very similar to those without a history of cancer. Patients with active cancer presented higher all-cause mortality, which may be related to cancer itself, but TAVR should not systematically be denied to these groups of patients. A comprehensive evaluation involving a multidisciplinary team of cardiologists and oncologists aimed to select candidates for TAVR given their higher risk of mortality in the mid- and long-term is desirable.

## Data Availability Statement

The raw data supporting the conclusions of this article will be made available by the authors, without undue reservation.

## Author Contributions

All authors listed have made a substantial, direct and intellectual contribution to the work, and approved it for publication.

## Conflict of Interest

The authors declare that the research was conducted in the absence of any commercial or financial relationships that could be construed as a potential conflict of interest.

## Publisher's Note

All claims expressed in this article are solely those of the authors and do not necessarily represent those of their affiliated organizations, or those of the publisher, the editors and the reviewers. Any product that may be evaluated in this article, or claim that may be made by its manufacturer, is not guaranteed or endorsed by the publisher.
