# Supplementary material for: Association Between Previous or Active Cancer and Clinical Outcomes in TAVR Patients: A Systematic Review and Meta-Analysis of 255,840 Patients
Source: Front Cardiovasc Med. 2021 Nov 2;8:763557. doi: 10.3389/fcvm.2021.763557 (PMC8593236; doi:10.3389/fcvm.2021.763557)
Supplement: Supplementary file 1 [file Data_Sheet_1.doc]

**SUPPLEMENTARY DATA**

**Association between previous or active cancer and clinical outcomes in TAVR patients: a systematic review and meta-analysis of 255,840 patients**

Diaz-Arocutipa C et al.

**Supplementary Table 1. Electronic search strategy (March 05, 2021)**

| **PubMed (139 hits)**  (TAVR[tiab] OR TAVI[tiab] OR “transcatheter aortic”[tiab] OR “percutaneous aortic”[tiab] OR “transcatheter aortic valve replacement”[mesh]) AND (cancer[tiab] OR malignanc*[tiab] OR neoplasm*[tiab] OR tumo*[tiab] OR neoplasms[mesh]) |
| --- |
| **EMBASE (305 hits)**  (tavr:ti,ab OR tavi:ti,ab OR 'transcatheter aortic':ti,ab OR 'percutaneous aortic':ti,ab) AND (cancer:ti,ab OR malignanc*:ti,ab OR neoplasm*:ti,ab OR tumo*:ti,ab) |
| **Scopus (368 hits)**  TITLE-ABS-KEY ((“TAVR” OR “TAVI” OR “transcatheter aortic” OR “percutaneous aortic”) AND (“cancer” OR “malignanc*” OR “neoplasm*” OR “tumo*”)) |
| **Web of Science (143 hits)**  TS=((“TAVR” OR “TAVI” OR “transcatheter aortic” OR “percutaneous aortic”) AND (“cancer” OR “malignanc*” OR “neoplasm*” OR “tumo*”)) |

**Supplementary Table 2. Main characteristics of cancer**

| **Study** | **Type of cancer** | **Staging of cancer** | **Treatment of cancer** |
| --- | --- | --- | --- |
| Watanabe, 2016 | Lungs (19%), prostate (15%), female breast (11%), colon and rectum (11%), liver (9%), ear-nose-throat (9%), stomach (4%), pancreas (4%), kidney (4%), bladder (4%), thyroid (4%), others | Not reported | Not reported |
| Agrawal, 2019 | Breast (44%), Hodgkin’s lymphoma (31%), lungs (15%), non-Hodgkin’s lymphoma (7%), others | Not reported | Not reported |
| Biancari, 2020 | Prostate (23%), breast (31%), colon (9%), lymphoma (8.4%), melanoma (5%), rectal (5%), bladder (4.6%), leukemia (2.4%), thyroid (1.9%), others | Not reported | Not reported |
| Grant, 2020 | Not reported | Not reported | Not reported |
| Guha, 2020 | Breast (19%), prostate (23%), lungs (7%), colon (10%), others (45%) | Not reported | Not reported |
| Jain, 2020 | Prostate (14%), breast (5%), leukemia/lymphoma (52%), lungs (7%), colorectal (3%), urinary/bladder (4%), uterine corpus (1%), others | Not reported | Not reported |
| Ghotra, 2020 | Prostate (19%), breast (15%), non-Hodgkin’s lymphoma (7%), lungs (61%), renal (28%), thyroid (18%) | Not reported | Not reported |
| Landes, 2019 | Gastrointestinal (22%), prostate (16%), hematological (16%), breast (15%), lungs (11%), urinary bladder (5%), renal (4%), melanoma (2%), others | Stage I (28%), stage II (21%), stage III (11%), stage IV (40%), metastasis (31%) | Antineoplastic therapy (29%), indication for oncological surgery (26%) |
| Lantelme, 2020 | Not reported | Not reported | Not reported |
| Lind, 2020 | Breast (25%), gastrointestinal (19%), prostate (16%), hematological (11%), urinary tract (10%), and skin cancer (10%) | Metastasis (6%) | History of surgery (77%), history of radiotherapy (24%), history of chemotherapy (34%), active chemotherapy (83%) |
| Mangner, 2017 | Male: prostate (42%), hematological (24%), colon (7%), female: breast (35%), hematological (30%), colon (18%) | Advanced cancer (37%), metastasis (29%) | Current cancer therapy (50%), watch-and-wait strategy (23%), paliative strategy (9%) |
| Romeo, 2020 | Not reported | Not reported | Not reported |
| Tabata, 2020 | Prostate (24%), breast (22%), colorectal (10%), bladder (6%), lung (4%), leukemia (8%) | Stage I (4%), stage II (18%), stage III (20%), stage IV (29%) | Surgery (72%), chemotherapy (29%), radiation therapy (30%) |

**Supplementary Table 3. Adjusted eff**ect estimates of the effect of cancer on all-cause mortality in TAVR patients

| **Study** | **Adjusted hazard ratio (95% CI)** | **Method of adjustment** | **Adjusted variables** |
| --- | --- | --- | --- |
| Agrawal, 2019 | 2.07 (1.27-3.38) | Cox proportional-hazards model | STS score, hyperlipidemia, COPD, end stage renal disease, pre- and post-hemoglobin, pre and post-creatinine, FEV1, right ventricular systolic pressure, and blood transfusion |
| Biancari, 2020 | 1.10 (0.89-1.36) | Cox proportional-hazards model | Age, eGFR, hemoglobin, sex, diabetes, pulmonary disease, oxygen therapy, atrial fibrillation, severe frailty, LVEF≤50%, recent acute heart failure, and transapical approach |
| Landes, 2019 | 2.37 (1.74-3.23) | Propensity-score matching with Cox proportional-hazards model | Age, sex, STS score, creatinine level, COPD, cerebrovascular events, anemia, hypertension, dyslipidemia, diabetes, percutaneous coronary intervention, myocardial infarction, frailty, and NYHA |
| Lind 1, 2020 | 1.47 (1.16-1.87) | Cox proportional-hazards model | Age, sex, STS score, pre-procedural pacemaker, peripheral artery disease, and NYHA |
| Lind 2, 2020 | 0.92 (0.66-1.29) |
| Mangner, 2017 | 2.10 (1.41-3.13) | Cox proportional-hazards model | BMI, NYHA III/IV, atrial fibrillation, indication (valve-in-valve vs native), myocardial infarction, stroke, renal failure, and bleeding |
| Tabata 1, 2020 | 1.56 (1.22-2.00) | Cox proportional-hazards model | BMI, eGFR, coronary artery disease, NYHA IV, COPD, C-reactive protein, LVEF, and paravalvular leakage post-TAVR≥2 |
| Tabata 2, 2020 | 1.38 (1.06-1.80) |

TAVR, transcatheter aortic valve replacement; CI, confidence interval; STS, Society of Thoracic Surgeons; COPD, chronic obstructive pulmonary disease; eGFR, estimated glomerular filtration rate; LVEF, left ventricular ejection fraction; NYHA, New York Heart Association; BMI, body mass index.

**Supplementary Table 4**. Newcastle-Ottawa scale for risk of bias assessment of cohort studies

| **Study** | **SELECTION** | | | | **COMPARABILITY** | **OUTCOME** | | | **Total (maximum = 9)** |
| --- | --- | --- | --- | --- | --- | --- | --- | --- | --- |
|  | **Representativeness of the exposed cohort** | **Selection of the non-exposed cohort** | **Ascertainment of the exposure** | **Outcome status at start of study** |  | **Assessment of the outcome** | **Length of follow-up** | **Adequacy of follow-up** |  |
| Watanabe, 2016 | * | * | * | * |  | * | * | * | 7 |
| Agrawal, 2019 | * | * | * | * | ** | * | * | * | 9 |
| Biancari, 2020 | * | * | * | * | ** | * | * | * | 9 |
| Grant, 2020 | * | * | * | * | ** | * |  | * | 8 |
| Guha, 2020 | * | * | * | * |  | * |  | * | 6 |
| Jain, 2020 | * | * | * | * | ** | * |  | * | 8 |
| Ghotra, 2020 | * | * | * | * | ** | * | * | * | 9 |
| Landes, 2019 | * | * | * | * | ** | * | * | * | 9 |
| Lantelme, 2020 | * | * | * | * | ** | * | * | * | 9 |
| Lind, 2020 | * | * | * | * | ** | * | * | * | 9 |
| Mangner, 2017 | * | * | * | * | ** | * | * | * | 9 |
| Romeo, 2020 | * | * | * | * |  | * | * | * | 7 |
| Tabata, 2020 | * | * | * | * | ** | * | * | * | 9 |


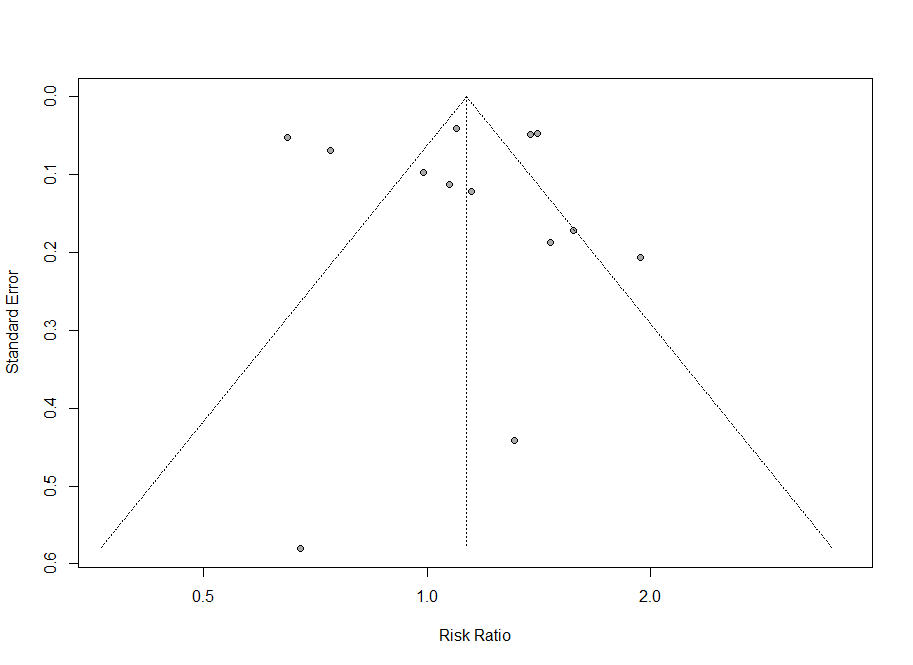


**Supplementary Figure 1. Funnel plot of all-cause mortality**
